# Sustainable Eating in Saudi Arabia: Associations Between Food Sustainability Knowledge, Attitudes, Food Waste-Related Behaviours, and Dietary Choices Among Adults

**DOI:** 10.3390/nu18071149

**Published:** 2026-04-03

**Authors:** Areej A. Alghamdi, Najlaa M. Aljefree, Israa M. Shatwan, Noha M. Almoraie

**Affiliations:** Department of Food and Nutrition, Faculty of Human Sciences and Design, King Abdulaziz University, Jeddah 22254, Saudi Arabia; aalghamdi3884@stu.kau.edu.sa (A.A.A.); naljefree@kau.edu.sa (N.M.A.); eshatwan@kau.edu.sa (I.M.S.)

**Keywords:** sustainable food habits, dietary choices, knowledge, attitude, behaviour

## Abstract

**Background/Objectives**: Sustainable food habits are essential for reducing the environmental impacts of a food system. We investigated food sustainability knowledge, attitudes, and food waste-related behaviours among Saudi adults and assessed their associations with socio-demographic characteristics and dietary choices, which are subjects that remain under-researched. **Methods**: A cross-sectional study was conducted among 855 Saudi adults (≥18 years) using convenience sampling. Data were collected using a validated online questionnaire assessing sustainability knowledge, attitudes, food waste behaviours, dietary choices, and socio-demographic characteristics. Descriptive statistics, chi-square tests, and linear regression analyses were performed using SPSS version 29. **Results**: Overall, 32% of the study population demonstrated adequate sustainability knowledge, 61% expressed positive attitudes towards food sustainability, and 45% demonstrated favourable food waste management. Women were more knowledgeable than men. Participants who possessed a better understanding of food sustainability consumed more vegetables, fruits, and bread and less processed meat. Those with a positive attitude towards food sustainability exhibited higher consumption of red meat, white meat, eggs, milk, yogurt, cheese, vegetables, fruits, bread, and sweet or savoury snacks. Meanwhile, individuals with better food waste behaviours demonstrated significantly lower consumption of legumes, fish, pasta, red meat, white meat, processed meat, eggs, milk, yogurt, cheese, fruits, bread, and sweet or savoury snacks. **Conclusions**: Saudi adults possess limited knowledge of sustainability and suboptimal food waste behaviours despite having relatively positive attitudes. These findings highlight opportunities for intervention. Public education, targeted campaigns, and media communication could enhance sustainability awareness and promote healthier, environmentally sustainable dietary patterns.

## 1. Introduction

The current global food system faces unprecedented challenges related to sustainability, environmental impact, and public health [1]. Notably, food systems account for ap-proximately 35% of total anthropogenic greenhouse gas (GHG) emissions, thereby exacerbating climate change [2]. Agricultural production accounts for 40% of global land use and 70% of freshwater withdrawals [3,4]. Furthermore, the transformation of natural ecosystems into farmland and pastureland remains a leading cause of biodiversity loss and soil degradation [5]. According to the EAT-Lancet Commission, food systems exert a major effect on Earth’s systems and represent the predominant cause of environmental deterioration [6]. Projections indicate that GHG emissions may increase by up to 51% by 2050 if current consumption patterns persist [7].

Several strategies, such as adopting healthier dietary patterns and reducing food loss and waste, can mitigate the environmental impacts of food systems; however, these measures require changes in consumption behaviour [6]. Dietary habits in developed countries, particularly meat consumption, substantially contribute to environmental degradation [8]. Transitioning towards diets lower in animal-based products and higher in plant-based foods is associated with considerable environmental benefits [9,10,11].

The Food and Agriculture Organisation defines sustainable diets as “diets with low environmental impacts which contribute to food and nutrition security and support healthy life for present and future generations. Sustainable diets are protective and respectful of biodiversity and ecosystems, culturally acceptable, accessible, economically fair and affordable; nutritionally adequate, safe and healthy, while optimising natural and human resources” [12]. Sustainable dietary patterns entail choices that provide health benefits, ensure food security, and minimise environmental impacts [6,13]. Accordingly, achieving sustainable food systems requires both modifications in food production and changes in consumer dietary behaviours and consumption patterns. The progression towards more sustainable diets necessitates a comprehensive understanding of the complex interrelationships among sustainability knowledge, attitudes towards sustainable eating, and actual dietary behaviours [14].

Within this framework, sustainability-related knowledge encompasses an individual’s understanding of the environmental, social, and economic implications of food production and consumption, whereas attitudes reflect evaluative perceptions regarding the importance of adopting sustainable food practices. These cognitive and perceptual dimensions interact with everyday behavioural patterns—such as food purchasing, storage, preparation, and disposal—which collectively influence food waste reduction. Together, these factors shape dietary choices aimed at mitigating environmental impacts while supporting human health [15,16,17].

Higher levels of sustainability knowledge and awareness are associated with greater engagement in sustainable eating behaviours, although discrepancies often exist between favourable attitudes and actual purchasing or consumption decisions [18]. Specifically, knowledge about sustainable diets affects dietary practices both directly and through mediating mechanisms, including personal motivations and practical behavioural strategies [19]. Sustainable food literacy, which integrates knowledge, food skills, attitudes, and action-based strategies, has emerged as a critical competency for promoting healthier and more environmentally responsible dietary choices [14]. However, evidence suggests that behavioural competencies—such as cooking skills and meal-planning abilities—may play an equally or even more significant role than attitudinal factors in translating sustainability values into actual eating practices [14].

Few studies have examined food sustainability awareness in Saudi Arabia. One study reported limited awareness and understanding of food sustainability and the environmental impacts of food production, despite evident interest in purchasing sustainable food products [20]. Another generational study revealed low awareness of sustainable, healthy diets, with observed generational differences in the consumption of animal- and plant-based protein [21]. Additionally, a separate investigation into climate change awareness found limited understanding but heightened concern, with greater awareness associated with more sustainable dietary behaviours [22].

Food waste constitutes a critical issue under the Sustainable Development Goals, having reached unprecedented levels, which, together with increasing consumption, contributes to higher domestic material consumption and a larger environmental footprint [23]. Approximately one-third of food produced for human consumption is either lost or wasted [24]. In Saudi Arabia, food waste is particularly salient owing to restricted agricultural resources and water supplies coupled with population growth, necessitating reliance on imported and subsidised food [25]. A nationwide cross-sectional study in Saudi Arabia reported high rates of household food waste (63.6% for uncooked food and 74.4% for cooked food), influenced by factors such as income, social support, household size, and the presence of older adults [26]. Additionally, regional studies have found that a substantial proportion of individuals discard leftovers, underscoring persistent food waste behaviours [27].

Despite growing global interest in sustainable food consumption, research on knowledge, attitudes, and behaviours related to food sustainability among Saudi adults remains limited. Specifically, little is known about the associations between these factors and various socio-demographic characteristics and dietary choices. To our knowledge, this is the first study in Saudi Arabia to simultaneously examine associations among food sustainability knowledge, attitudes towards food sustainability, food waste behaviours, and dietary choices in adults.

Accordingly, in this cross-sectional survey, we aimed to assess food sustainability knowledge, attitudes, and food waste-related behaviours among Saudi adults and to examine their associations with socio-demographic characteristics and dietary choices. Understanding these relationships is essential for developing effective strategies to promote sustainable and healthy eating practices.

## 2. Materials and Methods

### 2.1. Study Design

This cross-sectional survey was conducted to assess the food sustainability knowledge, attitudes, and food waste-related behaviours among Saudi adults and their associations with socio-demographic characteristics and dietary choices. This study was approved by the Biomedical Ethics Research Committee of King Abdulaziz University, Jeddah, Saudi Arabia (reference no 134-24). All participants were informed of the study objectives, the voluntary nature of participation, and data confidentiality. Informed consent was requested on the first page of the questionnaire. The survey was conducted in accordance with ethical research standards.

### 2.2. Study Participants and Data Collection

According to the most recent data provided by the General Authority for Statistics, the total Saudi population was 19,635,258 in 2024 [28]. Thus, the sample size required to achieve sufficient statistical power was 664, based on a 99% confidence interval (CI), 5% margin of error, and 50% response distribution [29]. The sample size was then increased to account for potential dropouts, yielding a final sample size of 797 participants. Individuals were recruited from the 13 administrative regions of Saudi Arabia. This study included Saudi nationals of both sexes, aged 18 years or older, residing within these regions. A non-probability convenience sampling method was employed, which, while potentially limiting generalisability, enabled the inclusion of a geographically diverse sample. The highest representation was from the Makkah (14%), Riyadh (12%), Al-Baha (12%), Al-Qassim (10%), Madinah (9%), and the Eastern Province (9%) regions. Data collection was conducted online using Google Forms as the survey platform. Recruitment was carried out via various social media platforms, including WhatsApp, Telegram, X, and Snapchat. Each invitation provided a brief overview detailing eligibility criteria, study objectives, estimated completion times, voluntary nature of participation, confidentiality assurances, and relevant contact information. Informed consent was requested on the first page of the questionnaire. Informed consent was obtained electronically, and only those who agreed to participate were permitted to proceed. A total of 855 responses were collected between June and November 2024.

### 2.3. Questionnaire Design

The questionnaire was adapted from that designed by Irazusta-Garmendia et al. [30], which was previously validated in a study conducted by García-González et al. on the Spanish population [31]. The questionnaire was translated into Arabic by a bilingual expert and back-translated into English by a blinded expert. The original English version, Arabic translation, and back-translated English version were compared to ensure that the original meaning and intent were retained while being linguistically and culturally appropriate for the Saudi population. A pilot version was distributed to 10 individuals (not included in the main study) who reviewed it for content, language, or layout issues. They were asked to provide feedback on how easy the questionnaire was to use, helping identify potential difficulties for future respondents. The feedback was incorporated into the final version of the questionnaire, and data from the pilot study were excluded from the analysis. This included the following:

1. Socio-demographic data: Nationality, age, sex, marital status, region of residence, educational attainment, occupation, and monthly income.
2. Knowledge of sustainability concepts: Participants were asked about their understanding of topics such as food sustainability, environmental footprint, carbon footprint, biodiversity, GHG emissions, water footprint, and food waste. The possible responses were ‘yes’, ‘no’, and ‘I have heard, but I do not know what it means’.
3. Priority assigned to various sustainable food attributes: Participants rated the importance of multiple attributes associated with sustainable diets, including low environmental impact, respect for biodiversity, no additives, minimal processing, fewer ingredients, organic production, abundance of fresh products, plant-based foods, locally sourced products, inclusion of traditional foods from their culture, accessibility, human health, and reduced food waste. Responses utilised a Likert scale: 0 (‘I do not know’), 1 (‘not important at all’), and 5 (‘very important’).
4. Impact of different types of foods on planetary sustainability: Participants were asked to indicate the impact of different food groups on sustainability, including plant-based foods, red meat, white meat, processed meat, fish and seafood, ultra-processed foods, vegetable oils, nuts, milk and dairy products, eggs, soft drinks, and ultra-processed beverages. Options included ‘positive impact’, ‘negative impact’, and ‘I do not know’.
5. Perceived importance of water use in food production: Participants were asked to identify the product that required the most water in food production. The possible responses were ‘plant-based products’, ‘animal-based products’, and ‘I do not know’.
6. Attitudes towards a sustainable diet: Participants were asked about the importance of sustainably produced products, their willingness to pay a premium for sustainably sourced food and beverages, and the importance of purchasing sustainable food options. Responses were recorded on a Likert scale from 1 (‘not important at all/not willing at all’) to 5 (‘very important/absolutely willing’).
7. Food waste behaviours: Participants were asked how frequently they left food uneaten on their plates, threw away spoiled food from the refrigerator or pantry, or wasted different food groups. The possible responses were ‘never’, ‘rarely’, ‘sometimes’, ‘often’, and ‘always’.
8. Food consumption frequency: Information on food consumption over the previous 12 months was collected using a validated 16-item food frequency questionnaire (FFQ) adapted from a previous study [30], focusing on foods selected for their environmental impact. Items were categorised as either high or low environmental impact. Sustainable diets are defined as those with a low environmental impact [12]. Foods with low environmental impact include legumes [32], fish [32,33], pasta [32,34], vegetables [32], fruits [32], and bread [35]. High-impact foods include red meat [32,35], white meat [35], processed meat [32,33], eggs [32,34], milk [35], yogurt [32,34], cheese [35], sweet or savoury snacks [33], sweetened soft drinks [33,36], and low-calorie soft drinks [33,36,37]. Consumption frequency options were never, 1–2 times per month, 1–2 times per week, 3–5 times per week, 1–2 times per day, and more than 3 times per day.

The food sustainability knowledge section comprised items 2−5, which were scored as follows. For item 2, a score of one point was assigned for ‘yes’ and 0 points for ‘no’ or ‘I heard, but I do not know what it means’. In item 3, 2 points were assigned for ‘very important’ or ‘important’, 1 point for ‘somewhat important’ or ‘less important’, and 0 points for ‘not important at all’ or ‘I do not know’. For item 4, 1 point was allocated for each correct identification of a ‘positive impact’ for foods with low environmental impact (i.e., plant-based foods [32], fish and seafood [32,33], vegetable oils [32], and nuts [32]). Similarly, 1 point was assigned for correctly selecting a ‘negative impact’ food with high environmental impact (e.g., red meat [32,35], white meat [35], processed meat [32,33], ultra-processed foods [38], milk and dairy products [32,34,35], eggs [32,34], soft drinks [33,36], and ultra-processed beverages [36]). However, incorrect answers and responses of ‘I do not know’ received 0 points. For item 5, 1 point was assigned for correctly selecting ‘animal-based products’, whereas 0 points were assigned for choosing ‘plant-based products’ or ‘I do not know’.

Furthermore, each item in the knowledge section was classified into two indicator levels: ‘high’ and ‘low’. For item 2, scores of 0–3 denoted ‘low knowledge’ and 4–7 indicated ‘high knowledge’. For item 3, scores of 0–13 indicated ‘low knowledge’, whereas 14–26 represented ‘high knowledge’. In item 4, a score of 0–6 was considered ‘low knowledge’, whereas 7–12 reflected ‘high knowledge’. For item 5, a score of 0 indicated ‘low knowledge’, and a score of 1 denoted ‘high knowledge’. Additionally, using a range of 4–8, scores of 4–5 were categorised as ‘low’ and scores of 6–8 as ‘high’.

The attitude towards food sustainability section comprised item 6, which was scored as follows: 2 points were assigned for ‘very important/absolutely willing’ and ‘important/willing’ responses, 1 point was assigned for ‘moderately important/moderately willing’, and 0 points were assigned for ‘little importance/unwilling’ and ‘not important at all/not willing at all’. Similarly, attitudes were classified into two levels: ‘negative’ (scores 0–3) and ‘positive’ (scores 4–6).

The food waste behaviours section comprised item 7 and was scored as follows: 2 points for ‘never’ or ‘rarely’, 1 point for ‘sometimes’, and 0 points for ‘often’ or ‘always’. Similarly, the food waste behaviours were classified into two indicator levels: ‘poor’ (scores 0–16) and ‘good’ (scores 17–32). The behaviour score assessed participants’ food waste practices. However, it reflected only food waste behaviours and does not constitute a comprehensive measure of environmentally sustainable dietary behaviour.

Cronbach’s alpha values for assessing the internal consistency reliability of the knowledge sections were 0.855, 0.941, and 0.911 for items 2, items 3, and items 4, respectively. Cronbach’s alpha for the attitude scale was 0.790, and for the behaviour scale, it was 0.908.

### 2.4. Statistical Analysis

Statistical analyses were performed using SPSS version 29 (IBM, Armonk, NY, USA). Participants’ socio-demographic characteristics as well as responses related to food sustainability knowledge, attitudes, and food waste-related behaviours are presented as frequencies and percentages. Data regarding participants’ knowledge, attitudes, food waste-related behaviours, and food consumption are summarised in Tables S1–S4. Scores corresponding to food sustainability knowledge, attitudes, and food waste-related behaviours are also reported as frequencies and percentages.

Chi-squared tests were used to examine associations between socio-demographic variables and food sustainability knowledge, attitudes, and food waste-related behaviours. Linear regression analyses further examined these associations, adjusting for covariates including sex, age group, marital status, education, occupation, and monthly income—selected based on their significant association with the knowledge, attitude, and behaviour scales. Food consumption scores were compared against food sustainability knowledge, attitudes, and food waste-related behaviour scores through adjusted linear regression analyses. A *p*-value < 0.05 was considered statistically significant.

## 3. Results

### 3.1. Socio-Demographic Characteristics

Table 1 summarises the socio-demographic characteristics of the 855 Saudi adult participants stratified by sex (405 men and 450 women). Most participants were aged 18–29 years (59%), were single (62%), held at least a bachelor’s degree or higher diploma (65%), and were students (49%). Approximately 30% of participants reported a monthly income between SAR 12,000 and 20,000. Significant sex differences were observed across all socio-demographic variables (*p* < 0.05). Men were more prevalent in the 50–59 years age group, whereas women were more prevalent in the 40–49 years age group. Single status was more common among men, whereas married and divorced/widowed statuses were more frequent among women. Additionally, men were more likely to have a basic education level, whereas women had higher postgraduate attainment. Occupational differences indicated a higher proportion of male students, private-sector employees, and retirees, whereas a greater proportion of females were not employed.

### 3.2. Factors Associated with Food Sustainability Knowledge, Attitudes, and Food Waste-Related Behaviours

Table 2 illustrates the associations between socio-demographic characteristics and participants’ food sustainability knowledge, attitudes, and food waste-related behaviours. Women demonstrated significantly higher knowledge levels than men (*p* < 0.001). Positive attitudes towards food sustainability were most prevalent among participants aged 18–29 years, single individuals, students, and those with a monthly income of SAR 12,000–20,000 (all *p* < 0.01). Additional analyses using tertile-based categorisation of food sustainability attitude scores showed similar patterns among socio-demographic groups (see Supplementary Table S9). Additionally, participants with a bachelor’s degree or higher demonstrated better food waste-related behaviours than those with basic or postgraduate education (*p* = 0.021). Overall, 32% of participants demonstrated high knowledge, 61% reported positive attitudes, and 45% exhibited good food waste behaviours.

Table 3 presents the sex-stratified associations between socio-demographic factors and food sustainability knowledge, attitudes, and food waste-related behaviours. Among women, knowledge scores were significantly associated with education level and occupation, with higher scores observed among those holding a bachelor’s degree or higher diploma and those working in freelance occupations (*p* < 0.05). Among men, attitude scores were positively associated with monthly income, with higher scores observed among those earning SAR 12,000 or more per month (*p* = 0.007).

### 3.3. Association Between Food Sustainability Knowledge, Attitudes, and Food Waste-Related Behaviours and Food Consumption

The participants’ intake of the selected food items according to their food sustainability knowledge scores is presented in Table 4. The participants were classified into low (n = 579) and high (n = 276) knowledge groups. Higher food sustainability knowledge scores were positively associated with the intake of white meat (*p* = 0.049), vegetables (*p* = 0.000), fruits (*p* = 0.000), and bread (*p* = 0.003). In contrast, knowledge scores were significantly negatively associated with processed meat intake when participants with high scores were compared to those with low scores (*p* = 0.025).

Table 5 presents participants’ intake of selected food items by food sustainability attitude scores, classified into low (n = 334) and high (n = 521) groups. Higher scores for attitudes towards food sustainability were positively associated with the intake of red meat (*p* = 0.000), white meat (*p* = 0.000), eggs (*p* = 0.000), milk (*p* = 0.001), yogurt (*p* = 0.000), cheese (*p* = 0.001), vegetables (*p* = 0.000), fruits (*p* = 0.001), bread (*p* = 0.000), and sweet or savoury snacks (*p* = 0.004). Tertile-based analyses of food sustainability attitude scores revealed comparable trends in dietary intake across the lowest and highest tertiles (Table S10).

Table 6 presents participants’ intake of selected food items by food waste-related behaviour scores, classified into low (n = 473) and high (n = 382) groups. Higher scores for food waste behaviours were negatively associated with the intake of legumes (*p* = 0.002), fish (*p* = 0.000), pasta (*p* = 0.000), red meat (*p* = 0.000), white meat (*p* = 0.000), processed meat (*p* = 0.000), eggs (*p* = 0.000), milk (*p* = 0.000), yogurt (*p* = 0.000), cheese (*p* = 0.000), fruits (*p* = 0.016), bread (*p* = 0.000), sweet or savoury snacks (*p* = 0.000), sweetened soft drinks (*p* = 0.000), and low-calorie soft drinks (*p* = 0.000).

## 4. Discussion

Understanding public knowledge, attitudes regarding food sustainability, and food waste-related behaviours is becoming increasingly important as global food systems face environmental and health challenges. Evidence indicates that sustainable dietary choices, including a higher consumption of plant-based foods, reduced food waste, and a preference for locally produced items, can mitigate climate change, conserve resources, and improve population health [6,39]. Previous studies have shown that knowledge and attitudes strongly influence engagement in sustainable food practices; however, these relationships vary by cultural and socio-demographic factors [22,31,40]. We assessed the food sustainability knowledge, attitudes, and food waste-related behaviours among Saudi adults, their associations with socio-demographic characteristics, and their relationship with dietary choices.

This study revealed limited knowledge, generally positive attitudes towards food sustainability, and suboptimal food waste-related behaviours among Saudi adults. These findings align with international and regional research indicating that limited awareness frequently coexists with favourable attitudes towards sustainable food choices. For instance, a study in Spain found that most participants lacked familiarity with the concepts and attributes of a sustainable diet, despite expressing positive attitudes towards adopting such practices [31]. In Turkey, only 30.5% of respondents were aware of the term ‘food sustainability ‘, yet their attitudes remained broadly supportive of sustainable eating [41]. Among young adults in Canada, although knowledge of sustainable diets varied, sustainability was a key motivator for dietary choices [42]. Another Spanish study found participants were more familiar with general aspects, such as food waste, with 60% reporting food waste at home [43]. In Kuwait, a limited understanding of key sustainability-related dietary factors including the environmental impact of certain food groups was observed, despite favourable views of sustainable diets [44].

Evidence from Saudi Arabia shows comparable trends. One study highlighted low levels of knowledge about food sustainability and limited recognition of the environmental consequences of food production yet reported positive attitudes towards sustainable food products [20]. Recent Saudi studies underscore this gap: only 24% of participants in one study reported familiarity with sustainable healthy diets [21], whereas another found nearly half (48.9%) were unaware of sustainable food options, despite showing a willingness to purchase local and organic products, even at higher prices [45].

In the current study, knowledge of food sustainability was significantly associated with sex, with women exhibiting higher scores than men. This disparity may reflect traditional gender roles in Saudi households, where women typically assume primary responsibility for meal planning, food purchasing, and meal preparation [46,47]. Additionally, generally higher educational attainment and interest in health-related information can enhance awareness of the environmental and intergenerational impacts of dietary choices [48,49,50]. These findings align with previous studies, although some have reported mixed results [20,31,41,44,51]. No significant association between sex and attitudes towards food sustainability was observed, aligning with earlier research [52]. The lack of sex differences in this area may be linked to nationwide initiatives, such as the Saudi Vision 2030 framework [53] and the Saudi Green Initiative [54], which aim to promote sustainability across all demographic groups and may have fostered similar positive attitudes among men and women.

In contrast, attitudes towards food sustainability were significantly associated with age, marital status, occupation, and monthly income, with younger participants showing more positive attitudes than older counterparts. This trend may result from generational differences in exposure to environmental issues. Younger generations, particularly Generation Z and Millennials, are being raised during a period when sustainability is prominently addressed in education, media, and public discourse, which may enhance their environmental awareness and pro-environmental attitudes [55,56]. As digital natives, they are also highly engaged with social media platforms that reinforce sustainability-related messages and purchasing behaviours [57,58,59]. Furthermore, their experiences during global crises, including the COVID-19 pandemic, may have heightened their concerns for both personal health and environmental well-being [57,60,61]. Conversely, older individuals who grew up when environmental concerns were less prominent tend to have more established dietary habits and values, which may limit their openness to adopting new sustainability-related behaviours [62]. For instance, in the United States, younger consumers express more positive attitudes towards sustainability and are willing to pay a premium for environmentally friendly products [63]; international research similarly identifies reduced interest among older cohorts [52], although a Spanish study reported that adults older than 50 years were more willing to pay for sustainable foods than younger adults [31]. In Saudi Arabia, older adults demonstrate more positive attitudes towards food sustainability, highlighting regional variations in generational perspectives [20]. Higher income levels were also positively associated with attitudes towards food sustainability. Comparable findings were observed in Kuwait, where individuals without income were less likely to hold positive attitudes towards sustainable diets than those with a monthly income [44]. Evidence from Slovakia demonstrates that households with higher incomes are willing to pay higher prices for local foods as sustainable choices than lower-income households [64]. According to Quoquab and Mohammad [65], the perceived or actual cost of sustainable consumption remains a barrier to widespread adoption.

Participants holding a bachelor’s degree or higher diploma demonstrated more favourable behaviours towards food waste than those with basic education and postgraduate degrees. This may be attributed to undergraduates’ younger age and tendency to reside with their families, resulting in less involvement in the purchasing, storage, and disposal of food. Those not responsible for household food management typically generate less food waste. A study in Thailand using a substantial national dataset of 2500 households revealed that higher educational levels correlate with improved food waste practices [66], and evidence from Lebanon indicates that higher education significantly decreases household food waste [67]. Higher education is associated with a better understanding of food labels, thereby enhancing one’s ability to manage food resources effectively. Conversely, findings from Morocco indicate that households led by individuals with higher educational attainment exhibit higher food waste, suggesting that other variables, such as income, lifestyle, and cultural norms, may have an important impact on food waste behaviours [68]. Consistent with this, the results of the current study indicate that postgraduate degree holders reported higher levels of food waste, potentially owing to life-stage factors, including time constraints and additional professional or familial responsibilities that may impede effective home food management [69].

Women engaged in freelance work exhibited the highest knowledge levels among all professional groups, likely attributable to the creative, digital, and cross-cultural characteristics of freelancing, which enhance exposure to global issues, including sustainability [70]. Despite initiatives under Saudi Vision 2030, food-related sustainability remains inadequately addressed. Male participants with higher incomes demonstrated more positive attitudes towards food sustainability, potentially owing to greater financial security and purchasing power, which mitigate cost-related barriers and facilitate sustainable dietary practices [71,72].

Analysis of associations between knowledge of and attitudes regarding food sustainability and corresponding food consumption patterns revealed that individuals with higher knowledge scores consumed greater amounts of white meat, vegetables, fruits, and bread, whereas they consumed less processed meat. Participants holding more positive attitudes tended to consume higher quantities of red meat, white meat, eggs, milk, yogurt, cheese, vegetables, fruits, bread, and sweet or savoury snacks. A previous study among students and health science professionals further supports these findings: individuals with greater knowledge and more favourable attitudes towards food sustainability reported an increased intake of fruits and vegetables and a decreased consumption of red and processed meats [30]. With the exception of red meat intake, our results align with these observations. Saudi Arabia is undergoing a rapid nutritional transition characterised by rising consumption of animal-source foods, urbanisation, and evolving food environments [21]. Furthermore, the social and symbolic significance of meat often associated with hospitality, affluence, and cultural tradition may partly explain the coexistence of positive sustainability attitudes and high animal product consumption observed in this study. These socio-cultural factors may moderate the relationship between sustainability attitudes and actual dietary behaviours. Moreover, limited awareness regarding the environmental consequences of red meat may perpetuate current consumption habits, as only 22% of respondents acknowledged the high water usage associated with animal products [73,74,75,76]. A review highlighted the need for substantial reductions in meat consumption, particularly in high-income countries, given the significant environmental, climatic, and health consequences of meat production compared with plant-based foods [77]. Evidence suggests that even minor reductions in meat intake (such as 100 g per week per individual) can substantially decrease GHG emissions, land use, and water consumption, underscoring the benefits of modest dietary shifts for natural resources and environmental health [78]. More substantial transitions toward plant-based diets have been associated with marked reductions in environmental impact, alongside significant health benefits, including decreasing the risks of obesity, type 2 diabetes, and cardiovascular disease [79].

Recent findings underscore a strong association between knowledge of sustainable diets and related dietary habits, with motivation identified as a crucial mediating factor [19]. Increased understanding of the environmental impacts of various meat types often fosters a preference for white meat. Empirical evidence demonstrates that white meat generally produces lower GHG emissions than red meat, particularly beef and lamb, which are characterised by high emissions owing to enteric fermentation in ruminants [9,80,81]. In addition, health considerations appear to be a primary driver of sustainable food choices, with individuals often prioritising foods perceived as beneficial to human health [82]. White meat is commonly viewed as a healthier option due to its lower saturated fat and cholesterol content and has been associated with more favourable health outcomes compared to red and processed meat [80,83,84,85,86]. Furthermore, foods with moderate environmental impacts, such as poultry, may serve as practical alternatives that support both health and sustainability goals, particularly when replacing higher-impact foods like red meat [32]. Economic considerations may also influence consumer behaviour, as white meat typically costs less than red meat. For instance, rising red meat prices in the United States have led many consumers to opt for more affordable alternatives such as chicken [87]. Evidence suggests that dietary shifts toward plant-based patterns, increased consumption of fresh and whole foods, and reduced food waste can improve both health outcomes and environmental sustainability [88]. Similarly, adherence to diets such as the Mediterranean diet has been associated with lower GHG emissions due to a reduced intake of animal-sourced foods [89]. However, in the present study, participants with more positive attitudes toward food sustainability reported a higher consumption of red meat, dairy products, eggs, and snacks. This apparent inconsistency suggests the presence of an attitude–behaviour gap, where favourable attitudes do not necessarily translate into sustainable dietary practices. A potential explanation is the lack of understanding and widespread misconceptions about sustainable dietary patterns; only 32% of participants demonstrated sufficient knowledge, which may result in an attitudinal–behavioural gap in which dietary choices do not align with sustainability goals. This gap may lead individuals to interpret sustainability primarily through a health-focused lens, without fully recognising its environmental dimensions [90]. Consequently, unclear or incomplete conceptualisation of sustainable diets may hinder the adoption of food choices aligned with sustainability principles. Studies have revealed that misconceptions regarding sustainable diets, primarily stemming from insufficient information, hinder their adoption [91]. Despite a growing awareness of the environmental impacts of consuming environmentally detrimental foods, dietary shifts often face resistance, hindered by ingrained cultural norms and personal preferences [92]. Despite university students’ intentions to adopt sustainable diets, significant challenges remain, including time constraints, the availability and cost of plant-based options, and the influence of family, partners, and peers [93]. In Saudi Arabia, traditional dietary practices centring on animal products may further impede progress toward sustainable dietary objectives. The socio-cultural and dietary context of Saudi Arabia may shape interpretations of food sustainability that diverge from Western low-carbon or plant-based paradigms; local priorities may include food freshness, sourcing, tradition, and overall quality, reflecting cultural norms and ongoing nutrition transitions [94].

Regarding the relationship between food waste behaviours and consumption, participants who demonstrated better food waste management reported a lower intake across nearly all categories, including both perishable items (e.g., fruits and dairy products) and non-perishable foods (e.g., legumes and soft beverages). This consistent pattern suggests that improved food management behaviours may be linked to more controlled and potentially less excessive food consumption. Participants with good food waste behaviour are likely to understand expiration dates, plan their meals in advance, control portion sizes, and manage leftovers effectively. These competencies serve to minimise food waste and reduce overconsumption. Food waste behaviours are also influenced by cultural and structural dynamics specific to Saudi Arabia, including family meal practices, food abundance, and norms regarding portioning and leftovers [25,47]. Previous research has confirmed that judicious selection and purchase of food quantities help prevent overconsumption and waste [95]. Findings from Italy confirm that young individuals who improve their food management practices and consumption generate less food waste [96]. Individuals with better food waste practices often perceive excessive consumption as problematic for religious, environmental, or health-related reasons. Thus, reduced overall consumption leads to diminished waste generation. Those who adopt waste-reducing practices support efforts to reduce the environmental impact of both food disposal and upstream activities in food production, processing, and distribution [97].

The findings of the current study can be interpreted using the Knowledge, Attitude, Behaviour model, which posits that individuals’ knowledge influences their attitudes, which in turn shape their behaviour. In this study, participants generally reported positive attitudes toward food sustainability; however, their comparatively limited knowledge of food sustainability and the presence of suboptimal food waste behaviours highlight a discrepancy between beliefs and actions. This pattern reflects the well-documented attitude–behaviour gap, in which individuals express support for food sustainability but do not consistently translate these attitudes into corresponding behaviours. Therefore, positive attitudes alone may be insufficient to promote behavioural change unless they are accompanied by adequate knowledge and favourable environmental factors.

This study presents several strengths. Few studies have concurrently evaluated food sustainability knowledge, attitudes, and food waste-related behaviours, particularly within Middle Eastern populations. Moreover, this study explored the associations between these constructs and dietary choices within the context of Saudi Arabia. Sixteen types of foods classified as environmentally friendly or otherwise based on previous literature were included. The sample size was relatively large and comprised only Saudi citizens. Nevertheless, certain limitations should be acknowledged. The reliance on self-reported data introduces several potential biases. The use of an FFQ, which required participants to accurately recall their dietary intake over the past 12 months, may have resulted in recall bias. Furthermore, as a cross-sectional study, causation can only be inferred indirectly. The application of non-probability convenience sampling may limit the generalisability of the findings to a broader population and could have introduced participation bias.

## 5. Conclusions

These findings suggest that food sustainability is a significant social concern within Saudi Arabia. The existence of positive attitudes even in the context of limited knowledge presents valuable opportunities for targeted intervention. However, the results also reveal a potential gap between favourable sustainability attitudes and actual dietary practices, suggesting that positive perceptions alone may not directly translate into more sustainable food choices. The findings emphasise the importance of strengthening sustainability-related knowledge and addressing behavioural and practical factors that influence daily food-related decisions. Specifically, strategies focused on improving food management skills, raising awareness of sustainable dietary practices, and minimising food waste are likely to be instrumental in promoting environmentally responsible eating behaviours. Furthermore, the results underscore the need to consider the broader socio-cultural landscape when analysing sustainable food behaviours, as cultural norms, traditional food practices, and perceptions of food quality and status may shape how sustainability concepts are understood and implemented in everyday diets. Strengthening public knowledge through educational initiatives, targeted campaigns, and media engagement could substantially improve both awareness and adoption of healthier, more environmentally sustainable dietary choices. Additionally, developing strategies that enhance the price competitiveness of sustainably produced foods and beverages is essential, as this can help attract lower-income consumers and reach broader segments of the population. Increasing public awareness of the environmental impact of food waste, particularly during the Hajj and Umrah seasons throughout Saudi Arabia, may also help reduce food waste, supporting the nation’s objectives under Vision 2030. This study offers critical insights for policymakers and researchers by identifying key demographic factors affecting food sustainability knowledge, attitudes, and related behaviours. Future research should consider employing quasi-experimental designs to further promote awareness of food sustainability and evaluate its impact on dietary habits, ultimately encouraging transitions towards more sustainable consumption patterns.

## Figures and Tables

**Table 1:** Socio-demographic characteristics of Saudi participants stratified by sex.

Characterisation	Total (n = 855) n (%)	Male (n = 405) n (%)	Female (n = 450) n (%)	X^2^	*p*-Value
Age (years)					
18–29	507 (59)	248 (61)	259 (58)	25.025	**<0.001**
30–39	144 (17)	65 (16)	79 (17)		
40–49	127 (15)	42 (11)	85 (19)		
50–59	57 (7)	41 (10)	16 (4)		
≥60	20 (2)	9 (2)	11 (2)		
Marital status					
Single	532 (62)	270 (67)	262 (58)	15.355	**<0.001**
Married	289 (34)	129 (32)	160 (36)		
Divorced/widowed	34 (4)	6 (1)	28 (6)		
Education					
Basic education levels	212 (25)	115 (28)	97 (22)	8.141	**0.017**
Bachelor’s degree/Higher diploma	556 (65)	258 (64)	298 (66)		
Postgraduate	87 (10)	32 (8)	55 (12)		
Occupation					
Student	415 (49)	209 (51)	206 (46)	69.599	**<0.001**
Government employed	191 (22)	93 (23)	98 (22)		
Private employed	69 (8)	44 (11)	25 (5)		
Free work	20 (2)	11 (3)	9 (2)		
Retired	39 (5)	29 (7)	10 (2)		
Unemployed	121 (14)	19 (5)	102 (23)		
Monthly income (SAR)					
<3000	129 (15)	60 (15)	69 (15)	19.852	**0.001**
3000–7000	176 (21)	61 (15)	115 (26)		
7000–12,000	188 (22)	90 (22)	98 (22)		
12,000–20,000	256 (30)	130 (32)	126 (28)		
>20,000	106 (12)	64 (16)	42 (9)		

Differences between groups were assessed using the chi-square test; *p* < 0.05 was considered significant. Bold values indicate statistically significant differences.

**Table 2:** Socio-demographic factors associated with Saudi participants’ food sustainability knowledge, attitudes, and food waste-related behaviours.

	Knowledge				Attitudes				Behaviours			
Socio-Demographic Characteristics	High Knowledge (n = 276) n (%)	Low Knowledge (n = 579) n (%)	X^2^	*p*-Value	Positive Attitude (n = 521) n (%)	Negative Attitude (n = 334) n (%)	X^2^	*p*-Value	Good Behaviour (n = 382) n (%)	Poor Behaviour (n = 473) n (%)	X^2^	*p*-Value
	Sex											
Male	104 (38)	301 (52)	15.341	**<0.001**	254 (49)	151 (45)	1.025	0.311	184 (48)	221 (47)	0.177	0.674
Female	172 (62)	278 (48)			267 (51)	183 (55)			198 (52)	252 (53)		
	Age (years)											
18–29	169 (61)	338 (58)	2.331	0.675	278 (53)	229 (69)	20.528	**<0.001**	232 (61)	275 (58)	7.205	0.125
30–39	40 (15)	104 (18)			101 (20)	43 (13)			68 (18)	76 (16)		
40–49	42 (15)	85 (15)			85 (16)	42 (13)			58 (15)	69 (15)		
50–59	17 (6)	40 (7)			42 (8)	15 (4)			16 (4)	41 (9)		
≥60	8 (3)	12 (2)			15 (3)	5 (1)			8 (2)	12 (2)		
	Marital status											
Single	167 (60)	365 (63)	0.837	0.658	302 (58)	230 (69)	10.434	**0.005**	246 (65)	286 (61)	1.621	0.445
Married	99 (36)	190 (33)			197 (38)	92 (27)			123 (32)	166 (35)		
Divorced/widowed	10 (4)	24 (4)			22 (4)	12 (4)			13 (3)	21 (4)		
	Education											
Basic education levels	66 (24)	146 (25)	0.524	0.769	120 (23)	92 (27)	2.643	0.267	110 (29)	102 (21)	7.737	**0.021**
Bachelor’s degree/Higher diploma	184 (67)	372 (64)			344 (66)	212 (64)			241 (63)	315 (67)		
Postgraduate	26 (9)	61 (11)			57 (11)	30 (9)			31 (8)	56 (12)		
	Occupation											
Student	132 (48)	283 (49)	5.125	0.401	226 (43)	189 (57)	20.628	**0.001**	200 (52)	215 (46)	5.207	0.391
Government employ	72 (26)	119 (21)			127 (24)	64 (19)			82 (22)	109 (23)		
Private employ	19 (7)	50 (8)			51 (10)	18 (5)			26 (7)	43 (9)		
Free work	8 (3)	12 (2)			15 (3)	5 (1)			9 (2)	11 (2)		
Retired	11 (4)	28 (5)			30 (6)	9 (3)			14 (4)	25 (5)		
Unemployed	34 (12)	87 (15)			72 (14)	49 (15)			51 (13)	70 (15)		
	Monthly income											
<3000	33 (12)	96 (17)	5.092	0.278	67 (13)	62 (19)	16.466	**0.002**	63 (16)	66 (14)	2.571	0.632
3000−7000	52 (19)	124 (21)			101 (19)	75 (22)			82 (22)	94 (20)		
7000−12,000	68 (24)	120 (21)			105 (20)	83 (25)			86 (22)	102 (21)		
12,000−20,000	88 (32)	168 (29)			178 (34)	78 (23)			106 (28)	150 (32)		
>20,000	35 (13)	71 (12)			70 (14)	36 (11)			45 (12)	61 (13)		

Differences between groups were assessed using the chi-square test; *p* < 0.05 was considered significant. Bold values indicate statistically significant differences.

**Table 3:** Association between food sustainability knowledge, attitudes, and food waste-related behaviours scores and Saudi participants’ socio-demographic characteristics stratified by sex.

Socio-Demographic	Male			Female		
	Knowledge	Attitudes	Behaviours	Knowledge	Attitudes	Behaviours
	Education					
Basic education levels	1.30 ± 0.45	1.57 ± 0.49	1.55 ± 0.50	1.33 ± 0.47	1.57 ± 0.49	1.48 ± 0.50
Bachelor’s degree/Higher diploma	1.23 ± 0.42	1.65 ± 0.47	1.42 ± 0.49	1.42 ± 0.49	1.59 ± 0.49	1.45 ± 0.49
Postgraduate	1.34 ± 0.48	1.66 ± 0.48	1.41 ± 0.49	1.27 ± 0.44	1.65 ± 0.48	1.33 ± 0.47
*p*-value	0.248	0.248	0.085	**0.024**	0.512	0.082
	Occupation					
Student	1.27 ± 0.44	1.52 ± 0.50	1.50 ± 0.50	1.37 ± 0.48	1.57 ± 0.49	1.47 ± 0.50
Government employed	1.28 ± 0.45	1.69 ± 0.46	1.47 ± 0.50	1.47 ± 0.50	1.64 ± 0.48	1.39 ± 0.49
Private employed	1.20 ± 0.40	1.82 ± 0.39	1.36 ± 0.48	1.40 ± 0.50	1.60 ± 0.50	1.40 ± 0.50
Free work	1.09 ± 0.30	1.82 ± 0.40	1.27 ± 0.46	1.78 ± 0.44	1.67 ± 0.50	1.67 ± 0.50
Retired	1.21 ± 0.41	1.79 ± 0.41	1.31 ± 0.47	1.50 ± 0.52	1.70 ± 0.48	1.50 ± 0.52
Unemployed	1.32 ± 0.47	1.68 ± 0.47	1.42 ± 0.50	1.27 ± 0.44	1.58 ± 0.49	1.42 ± 0.49
*p*-value	0.607	0.195	0.464	**0.027**	0.988	0.621
	Monthly income					
<3000	1.17 ± 0.37	1.45 ± 0.50	1.48 ± 0.50	1.33 ± 0.47	1.58 ± 0.49	1.49 ± 0.50
3000−7000	1.31 ± 0.46	1.57 ± 0.49	1.59 ± 0.49	1.29 ± 0.45	1.57 ± 0.49	1.40 ± 0.49
7000−12,000	1.29 ± 0.45	1.58 ± 0.49	1.42 ± 0.49	1.43 ± 0.49	1.54 ± 0.50	1.49 ± 0.50
12,000−20,000	1.24 ± 0.42	1.77 ± 0.42	1.41 ± 0.49	1.45 ± 0.50	1.62 ± 0.48	1.42 ± 0.49
>20,000	1.28 ± 0.45	1.63 ± 0.48	1.44 ± 0.50	1.40 ± 0.49	1.71 ± 0.45	1.40 ± 0.49
*p*-value	0.379	**0.007**	0.425	0.091	0.501	0.569

Linear regression was used to examine the association between socio-demographic characteristics and food sustainability knowledge, attitudes, and food waste-related behaviours scores; *p* < 0.05 was considered significant. Bold values indicate statistically significant differences.

**Table 4:** Intake of selected food items according to knowledge of food sustainability score.

Food Item	Low Score (n = 579)	High Score (n = 276)	Beta Coefficient (95% CI) *	Adjusted *p*-Value
Legumes	1.46 ± 1.05	1.61 ± 0.01	−0.14 (−0.30–0.002)	0.053
Fish	1.09 ± 0.90	1.06 ± 0.79	0.02 (−0.10–0.15)	0.689
Pasta	1.81 ± 0.98	1.87 ± 0.94	−0.02 (−0.17–0.11)	0.691
Red meat	1.93 ± 1.16	1.89 ± 0.10	0.01 (−0.15–0.17)	0.941
White meat	2.41 ± 1.26	2.58 ± 1.01	−0.17 (−0.34 to −0.001)	**0.049**
Processed meat	0.92 ± 1.18	0.71 ± 0.96	0.18 (0.02–0.34)	**0.025**
Eggs	2.44 ± 1.24	2.54 ± 1.22	−0.11 (−0.29–0.06)	0.209
Milk	2.42 ± 1.28	2.45 ± 1.23	−0.01 (−0.20–0.16)	0.871
Yogurt	2.34 ± 1.19	2.43 ± 1.20	−0.06 (−0.23–0.10)	0.459
Cheese	2.46 ± 1.26	2.63 ± 1.21	−0.15 (−0.33–0.02)	0.096
Vegetables	2.36 ± 1.21	2.89 ± 1.29	−0.49 (−0.67 to −0.32)	**0.000**
Fruits	2.16 ± 1.16	2.59 ± 1.29	−0.37 (−0.54 to −0.19)	**0.000**
Bread	2.77 ± 1.38	3.07 ± 1.24	−0.29 (−0.48 to −0.09)	**0.003**
Sweet or savoury snacks	2.27 ± 1.31	2.44 ± 1.23	−0.16 (−0.34–0.02)	0.089
Sweetened soft drinks	1.92 ± 1.39	1.74 ± 1.39	0.12 (−0.07–0.32)	0.227
Low-calorie soft drinks	1.43 ± 1.12	1.43 ± 1.31	−0.03 (−0.22–0.15)	0.744

*p*-value was analysed using univariate regression adjusted for sex, age group, marital status, education, occupation, and monthly income; *p* < 0.05 was considered significant. * High score considered as reference. CI: confidence interval. Bold values indicate statistically significant differences.

**Table 5:** Intake of selected food items according to attitudes of food sustainability score.

Food Item	Low Score (n = 334)	High Score (n = 521)	Beta Coefficient (95% CI) *	Adjusted *p*-Value
Legumes	1.42 ± 1.07	1.56 ± 1.01	−0.12 (−0.26–0.02)	0.102
Fish	1.09 ± 0.87	1.08 ± 0.86	0.04 (−0.07–0.16)	0.465
Pasta	1.81 ± 1.02	1.84 ± 0.93	−0.03 (−0.17–0.09)	0.581
Red meat	1.65 ± 1.09	2.08 ± 1.14	−0.37 (−0.53 to −0.22)	**0.000**
White meat	2.13 ± 1.20	2.69 ± 1.13	−0.50 (−0.66 to −0.34)	**0.000**
Processed meat	0.93 ± 1.10	0.80 ± 1.13	0.12 (−0.03–0.27)	0.129
Eggs	2.19 ± 1.22	2.65 ± 1.21	−0.40 (−0.57 to −0.23)	**0.000**
Milk	2.22 ± 1.27	2.57 ± 1.25	−0.31 (−0.48 to −0.13)	**0.001**
Yogurt	2.16 ± 1.19	2.51 ± 1.17	−0.31 (−0.47 to −0.14)	**0.000**
Cheese	2.31 ± 1.24	2.65 ± 1.23	−0.31 (−0.47 to −0.13)	**0.001**
Vegetables	2.31 ± 1.26	2.67 ± 1.24	−0.33 (−0.50 to −0.16)	**0.000**
Fruits	2.11 ± 1.19	2.42 ± 1.22	−0.28 (−0.44 to −0.11)	**0.001**
Bread	2.59 ± 1.35	3.04 ± 1.30	−0.39 (−0.58 to −0.21)	**0.000**
Sweet or savoury snacks	2.16 ± 1.23	2.42 ± 1.31	−0.26 (−0.43 to −0.08)	**0.004**
Sweetened soft drinks	1.81 ± 1.30	1.90 ± 1.45	−0.11 (−0.29–0.08)	0.274
Low-calorie soft drinks	1.55 ± 1.27	1.36 ± 1.33	0.14 (−0.04–0.32)	0.132

*p*-value was analysed using univariate regression adjusted for sex, age group, marital status, education, occupation, and monthly income; *p* < 0.05 was considered significant. * High score considered as reference. CI: confidence interval. Bold values indicate statistically significant differences.

**Table 6:** Intake of selected food items according to behaviours related to food waste score.

Food Item	Low Score (n = 473)	High Score (n = 382)	Beta Coefficient (95% CI) *	Adjusted *p*-Value
Legumes	1.62 ± 1.03	1.37 ± 1.03	0.22 (0.002–0.08)	**0.002**
Fish	1.21 ± 0.88	0.93 ± 0.82	0.27 (0.15–0.38)	**0.000**
Pasta	1.99 ± 0.96	1.63 ± 0.94	0.36 (0.23–0.49)	**0.000**
Red meat	2.09 ± 1.10	1.70 ± 1.15	0.37 (0.22–0.52)	**0.000**
White meat	2.65 ± 1.06	2.24 ± 1.30	0.37 (0.21–0.53)	**0.000**
Processed meat	1.00 ± 1.22	0.66 ± 0.94	0.34 (0.19–0.49)	**0.000**
Eggs	2.67 ± 1.14	2.22 ± 1.30	0.41 (0.25–0.58)	**0.000**
Milk	2.62 ± 1.18	2.20 ± 1.33	0.39 (0.21–0.56)	**0.000**
Yogurt	2.55 ± 1.09	2.15 ± 1.27	0.35 (0.19–0.51)	**0.000**
Cheese	2.77 ± 1.13	2.20 ± 1.30	0.54 (0.37–0.71)	**0.000**
Vegetables	2.62 ± 1.15	2.42 ± 1.38	0.16 (−0.01–0.33)	0.060
Fruits	2.41 ± 1.16	2.17 ± 1.29	0.20 (0.03–0.36)	**0.016**
Bread	3.08 ± 1.25	2.60 ± 1.41	0.44 (0.26–0.62)	**0.000**
Sweet or savoury snacks	2.54 ± 1.18	2.06 ± 1.35	0.46 (0.29–0.63)	**0.000**
Sweetened soft drinks	2.11 ± 1.40	1.55 ± 1.31	0.55 (0.37–0.73)	**0.000**
Low-calorie soft drinks	1.57 ± 1.36	1.26 ± 1.23	0.34 (0.16–0.52)	**0.000**

*p*-value was analysed using univariate regression adjusted for sex, age group, marital status, education, occupation, and monthly income; *p* < 0.05 was considered significant. * High score considered as reference. CI: confidence interval. Bold values indicate statistically significant differences.

## Data Availability

The data presented in this study are available on request from the corresponding author as the datasets generated and/or analysed during this study are not publicly available owing to the use of data for further publications.

## Supplementary Materials

The following supporting information can be downloaded at: https://www.mdpi.com/article/10.3390/nu18071149/s1, Table S1. Participants’ knowledge of food sustainability; Table S2. Participants’ attitudes toward food sustainability; Table S3. Participants’ food waste-related behaviours; Table S4. Participants’ frequency of consumption of different food groups. Table S5. Beta coefficients (95% confidence intervals) for sustainability knowledge, attitudes, and food waste-related behaviours and Saudi participants’ socio-demographic characteristics stratified by sex. Table S6. Association between food sustainability knowledge scores (continuous) and Saudi participants’ socio-demographic characteristics stratified by sex. Table S7. Association between food sustainability attitude scores (continuous) and Saudi participants’ socio-demographic characteristics stratified by sex. Table S8. Association between food waste behaviour scores (continuous) and Saudi participants’ socio-demographic characteristics stratified by sex. Table S9. Socio-demographic factors associated with tertiles of food sustainability attitude scores among Saudi participants. Table S10. Intake of selected food items according to tertiles of food sustainability attitude scores.

## Abbreviations

The following abbreviations are used in this manuscript:

CI	Confidence interval
FFQ	Food frequency questionnaire
GHG	Greenhouse gas
