# Supplementary material for: Sustainable Eating in Saudi Arabia: Associations Between Food Sustainability Knowledge, Attitudes, Food Waste-Related Behaviours, and Dietary Choices Among Adults
Source: Nutrients. 2026 Apr 3;18(7):1149. doi: 10.3390/nu18071149 (PMC13075233; doi:10.3390/nu18071149)
Supplement: Supplementary file 1 [file nutrients-18-01149-s001.zip › nutrients-4157487-supplementary.pdf]

Table S1. Participants’ knowledge of food sustainability.

| **Knowledge questions** | **Total (n = 855)** *n* (%) | | |
|---|---|---|---|
| **Are you familiar with the following concepts?** | **No** | **Yes** | **I heard of the term but, I do not know what it means** |
| Food sustainability | 293 (34) | 285 (33) | 277 (33) |
| Ecological footprint | 477 (56) | 165 (19) | 213 (25) |
| Carbon footprint | 480 (56) | 118 (14) | 257 (30) |
| Biodiversity | 287 (34) | 334 (39) | 234 (27) |
| Greenhouse gases emissions | 368 (43) | 248 (29) | 239 (28) |
| Water footprint | 528 (62) | 112 (13) | 215 (25) |
| Food waste | 128 (15) | 543 (64) | 184 (22) |

| **To what extent do you consider that each of the following aspects contribute to sustainable diet?** | **I do not know** | **Not important at all** | **Less important** | **Somewhat important** | **Important** | **Very important** |
|---|---|---|---|---|---|---|
| Low environmental impact | 355 (42) | 61 (7) | 118 (14) | 188 (22) | 72 (8) | 61 (7) |
| Respectful of biodiversity | 277 (32) | 60 (7) | 96 (11) | 210 (25) | 107 (13) | 105 (12) |
| No additives | 142 (17) | 64 (7) | 88 (10) | 237 (28) | 104 (12) | 220 (26) |
| Minimally processed products | 175 (21) | 61 (7) | 93 (11) | 220 (26) | 124 (14) | 182 (21) |
| Few ingredients | 263 (31) | 127 (15) | 157 (18) | 173 (20) | 69 (8) | 66 (8) |
| Organic food production | 166 (20) | 72 (8) | 77 (9) | 211 (25) | 96 (11) | 233 (27) |
| Diet with plenty of fresh products | 183 (22) | 53 (6) | 73 (9) | 209 (24) | 113 (13) | 224 (26) |
| Diet rich in plant-based foods | 185 (22) | 66 (8) | 86 (10) | 210 (24) | 136 (16) | 172 (20) |
| Locally grown products | 192 (22) | 86 (10) | 102 (12) | 204 (24) | 109 (13) | 162 (19) |
| Diet with traditional foods from own culture | 216 (25) | 110 (13) | 120 (14) | 209 (25) | 97 (11) | 103 (12) |
| Accessible | 191 (22) | 71 (8) | 94 (11) | 198 (23) | 126 (15) | 175 (21) |
| Healthy for humans | 128 (15) | 55 (6) | 68 (8) | 202 (24) | 121 (14) | 281 (33) |
| Reducing food waste | 275 (32) | 124 (14) | 91 (11) | 156 (18) | 76 (9) | 133 (16) |

| **Indicate the impact that you think the following foods have on the sustainability of the planet** | **I do not know** | **Negative impact** | **Positive impact** |
|---|---|---|---|
| Plant-based food | 181 (21) | 519 (61) | 155 (18) |
| Red meat | 145 (17) | 382 (45) | 328 (38) |
| White meat | 139 (16) | 331 (39) | 385 (45) |
| Processed meat | 184 (21) | 390 (46) | 281 (33) |
| Fish and seafood | 174 (20) | 318 (37) | 363 (43) |
| Ultra-processed foods | 180 (21) | 423 (50) | 252 (29) |
| Vegetable oils | 185 (22) | 380 (44) | 290 (34) |
| Nuts | 212 (25) | 452 (53) | 191 (22) |
| Milk and dairy products | 144 (17) | 305 (36) | 406 (47) |
| Eggs | 155 (18) | 286 (34) | 414 (48) |

| | | | |
|---|---|---|---|
| Soft drinks | 179 (21) | 403 (47) | 273 (32) |
| Ultra-processed beverages | 205 (24) | 324 (38) | 326 (38) |

| **Which product do you think needs more water for its production?** | **I do not know** | **Plant-based products** | **Animal-based products** |
|---|---|---|---|
| | 130 (15) | 539 (63) | 186 (22) |

Table S2. Participants' attitudes toward food sustainability.

| **Attitude questions** | **Total (n = 855)** *n* **(%)** | | | | |
|---|---|---|---|---|---|
| **How important is it for you that the products you consume be sustainably produced?** | **Not important at all** | **Little Importance** | **Moderately Important** | **Important** | **Very important** |
| | 66 (8) | 54 (6) | 198 (23) | 155 (18) | 382 (45) |
| **To what extent would you be willing to pay more money for sustainably produced food and beverages?** | **Not willing at all** | **Unwilling** | **Moderately willing** | **Willing** | **Absolutely willing** |
| | 80 (9) | 129 (15) | 300 (35) | 180 (21) | 166 (20) |
| **How important is it for you to buy sustainable food?** | **Not important at all** | **Little Importance** | **Moderately Important** | **Important** | **Very important** |
| | 68 (8) | 95 (11) | 273 (32) | 187 (22) | 232 (27) |

Table S3. Participants' food waste-related behaviours.

| **Food waste behaviours questions** | **Total (n = 855)** *n* **(%)** | | | | |
|---|---|---|---|---|---|
| **State how often you leave food on your plate** | **Never** | **Rarely** | **Sometimes** | **Often** | **Always** |
| | 44 (5) | 95 (11) | 321 (38) | 277 (32) | 118 (14) |
| **State how often you throw away spoiled food from the refrigerator or pantry** | **Never** | **Rarely** | **Sometimes** | **Often** | **Always** |
| | 36 (4) | 41 (5) | 182 (21) | 249 (29) | 347 (41) |
| **State how often you waste the following foods** | **Never** | **Rarely** | **Sometimes** | **Often** | **Always** |
| Legumes | 173 (20) | 135 (16) | 296 (35) | 187 (22) | 64 (7) |
| Fish | 190 (22) | 252 (30) | 240 (28) | 127 (15) | 46 (5) |
| Pasta | 87 (10) | 209 (24) | 252 (30) | 210 (25) | 97 (11) |
| Red meat | 103 (12) | 235 (27) | 272 (32) | 169 (20) | 76 (9) |
| White meat | 82 (10) | 219 (26) | 233 (27) | 216 (25) | 105 (12) |
| Processed meat | 344 (40) | 124 (15) | 159 (19) | 148 (17) | 80 (9) |
| Eggs | 89 (10) | 304 (35) | 219 (26) | 161 (19) | 82 (10) |
| Milk | 84 (10) | 239 (28) | 229 (27) | 199 (23) | 104 (12) |

| | | | | | |
|---|---|---|---|---|---|
| Yogurt | 87 (10) | 250 (29) | 208 (24) | 188 (22) | 122 (15) |
| Cheese | 84 (10) | 227 (26) | 214 (25) | 193 (23) | 137 (16) |
| Vegetables | 78 (9) | 156 (18) | 199 (23) | 233 (28) | 189 (22) |
| Fruit | 69 (8) | 164 (19) | 210 (25) | 227 (26) | 185 (22) |
| Bread | 64 (8) | 152 (18) | 217 (25) | 243 (28) | 179 (21) |
| Sweet or savoury snacks | 106 (12) | 239 (28) | 239 (28) | 180 (21) | 91 (11) |

Table S4. Participants' frequency of consumption of different food groups.

| **Dietary habits question** | **Total (n = 855)** ***n*** **(%)** | | | | | |
|---|---|---|---|---|---|---|
| **State how often you consume the following foods** | **Never** | **1–2 Times a Month** | **1–2 Times a Week** | **3–5 Times a Week** | **1–2 Times a Day** | **> 3 Times a Day** |
| Legumes | 149 (17) | 296 (35) | 269 (32) | 115 (13) | 21 (2) | 5 (1) |
| Fish | 205 (24) | 445 (52) | 144 (17) | 51 (6) | 9 (1) | 1 (0) |
| Pasta | 78 (9) | 221 (26) | 357 (42) | 173 (20) | 21 (2) | 5 (1) |
| Red meat | 99 (12) | 225 (26) | 246 (29) | 230 (27) | 44 (5) | 11 (1) |
| White meat | 76 (9) | 98 (11) | 193 (23) | 341 (40) | 132 (15) | 15 (2) |
| Processed meat | 453 (53) | 196 (23) | 121 (14) | 61 (7) | 16 (2) | 8 (1) |
| Eggs | 73 (9) | 109 (13) | 208 (24) | 302 (35) | 134 (16) | 29 (3) |
| Milk | 80 (9) | 108 (12) | 229 (27) | 271 (32) | 133 (16) | 34 (4) |
| Yogurt | 66 (8) | 126 (15) | 249 (29) | 282 (33) | 104 (12) | 28 (3) |
| Cheese | 66 (7) | 101 (12) | 230 (27) | 281 (33) | 137 (16) | 40 (5) |
| Vegetables | 61 (7) | 101 (12) | 246 (29) | 282 (33) | 101 (12) | 64 (7) |
| Fruit | 62 (7) | 154 (18) | 271 (32) | 245 (29) | 79 (9) | 44 (5) |
| Bread | 60 (7) | 75 (9) | 164 (19) | 265 (31) | 202 (24) | 89 (10) |
| Sweet or savoury snacks | 80 (9) | 147 (17) | 233 (27) | 247 (29) | 108 (13) | 40 (5) |
| Sweetened soft drinks | 175 (20) | 190 (22) | 219 (26) | 153 (18) | 86 (10) | 32 (4) |
| Low-calorie soft drinks | 286 (34) | 181 (21) | 191 (22) | 141 (16) | 43 (5) | 13 (2) |

Table S5. Beta coefficients (95% CI) for food sustainability knowledge, attitudes, and food waste-related behaviours and Saudi participants' socio-demographic characteristics, stratified by sex.

| Socio-demographic | Male | | | Female | | |
|---|---|---|---|---|---|---|
| | **Knowledge** | **Attitudes** | **Behaviours** | **Knowledge** | **Attitudes** | **Behaviours** |
| Age (years) | | | | | | |
| 18–29 | -0.06 (-0.43-0.30) | -0.29 (-0.69-0.09) | -0.02 (-0.44-0.38) | 0.01 (-0.29-0.33) | -0.08 (-0.40-0.23) | -0.06 (-0.38-0.26) |
| 30–39 | -0.21 (-0.53-0.11) | -0.10 (-0.45-0.25) | -0.05 (-0.31-0.42) | -0.01 (-0.33-0.29) | -0.01 (-0.32-0.31) | 0.05 (-0.26-0.37) |
| 40–49 | -0.23 (-0.56-0.09) | -0.15 (-0.51-0.21) | 0.06 (-0.31-0.43) | -0.001 (-0.31-0.31) | -0.01 (-0.33-0.30) | 0.01 (-0.31-0.33) |
| 50–59 | -0.21 (-0.54-0.11) | -0.09 (-0.44-0.25) | -0.09 (-0.45-0.27) | 0.10 (-0.27-0.48) | -0.08 (-0.47-0.29) | -0.15 (-0.54-0.23) |
| ≥ 60 | Reference | Reference | Reference | Reference | Reference | Reference |
| p-value | 0.199 | 0.262 | 0.522 | 0.921 | 0.849 | 0.435 |
| Marital status | | | | | | |
| Single | 0.16 (-0.21-0.54) | -0.08 (-0.48-0.32) | 0.21 (-0.21-0.62) | -0.01 (-0.22-0.19) | -0.02 (-0.24-0.18) | -0.002 (-0.21- 0.21) |
| Married | 0.26 (-0.09-0.63) | -0.10 (-0.49-0.28) | 0.22 (-0.18-0.63) | 0.01 (-0.18-0.21) | -0.002 (-0.20- 0.20) | 0.05 (-0.15-0.25) |
| Divorced/ widowed | Reference | Reference | Reference | Reference | Reference | Reference |
| p-value | 0.194 | 0.858 | 0.551 | 0.904 | 0.920 | 0.712 |
| Education | | | | | | |
| Basic education levels | -0.03 (-0.21-0.14) | 0.01 (-0.18-0.19) | 0.11 (-0.08-0.31) | 0.11 (-0.05-0.27) | -0.09 (-0.26-0.07) | 0.18 (0.01-0.35) |
| Bachelor's degree /Higher diploma | -0.10 (-0.27-0.07) | 0.08 (-0.09-0.27) | -0.01 (-0.20-0.18) | 0.18 (0.04-0.33) | -0.08 (-0.22-0.06) | 0.15 (0.004-0.30) |
| Postgraduate | Reference | Reference | Reference | Reference | Reference | Reference |
| p-value | 0.248 | 0.248 | 0.085 | **0.024** | 0.512 | 0.082 |
| Occupation | | | | | | |
| Student | -0.05 (-0.29-0.15) | -0.15 (-0.38-0.06) | 0.04 (-0.19-0.28) | 0.05 (-0.08-0.19) | 0.01 (-0.12-0.15) | 0.08 (-0.05-0.22) |
| Government employed | -0.08 (-0.32-0.15) | -0.09 (-0.35-0.15) | 0.15 (-0.11-0.42) | 0.20 (0.05-0.35) | 0.01 (-0.14-0.16) | -0.02 (-0.17-0.13) |
| Private employed | -0.15 (-0.40-0.09) | 0.05 (-0.21-0.32) | 0.01 (-0.26-0.29) | 0.08 (-0.13-0.30) | 0.04 (-0.18-0.26) | -0.01 (-0.23-0.22) |
| Free work | -0.27 (-0.61-0.07) | 0.02 (-0.34-0.39) | -0.06 (-0.44-0.32) | 0.44 (0.11-0.77) | 0.11 (-0.23-0.45) | 0.23 (-0.10-0.58) |
| Retired | -0.16 (-0.47-0.15) | -0.07 (-0.41-0.26) | 0.001 (-0.35-0.35) | 0.25 (-0.09-0.61) | 0.07 (-0.29-0.43) | 0.01 (-0.34-0.38) |
| Unemployed | Reference | Reference | Reference | Reference | Reference | Reference |
| p-value | 0.607 | 0.195 | 0.464 | **0.027** | 0.988 | 0.621 |
| Monthly income | | | | | | |

| | | | | | | |
|---|---|---|---|---|---|---|
| < 3,000 | -0.11 (-0.27-0.04) | -0.12 (-0.29-0.05) | 0.01 (-0.17-0.19) | -0.07 (-0.26-0.11) | -0.11 (-0.31-0.08) | 0.07 (-0.12-0.26) |
| 3,000–7,000 | 0.02 (-0.13-0.18) | -0.001 (-0.17-0.17) | 0.10 (-0.07-0.29) | -0.12 (-0.30-0.04) | -0.11 (-0.29-0.06) | -0.02 (-0.21-0.15) |
| 7,000–12,000 | 0.01 (-0.13-0.15) | -0.03 (-0.18-0.12) | -0.03 (-0.20-0.12) | 0.01 (-0.16-0.19) | -0.16 (-0.34-0.01) | 0.06 (-0.11-0.24) |
| 12,000–20,000 | -0.04 (-0.17-0.09) | 0.14 (0.001-0.28) | -0.03 (-0.18-0.11) | 0.03 (-0.13-0.21) | -0.09 (-0.26-0.08) | 0.002 (-0.17-0.17) |
| >20,000 | Reference | Reference | Reference | Reference | Reference | Reference |
| p-value | 0.379 | **0.007** | 0.425 | 0.091 | 0.501 | 0.569 |

CI: confidence interval

Table S6. Association between food sustainability knowledge scores (continuous) and Saudi participants' socio-demographic characteristics, stratified by sex.

| | Male | | Female | |
|---|---|---|---|---|
| **Socio-demographic** | **Mean ± SD** | **Beta coefficients (95% CI)** | **Mean ± SD** | **Beta coefficients (95% CI)** |
| | | Age (years) | | |
| 18–29 | 18.04± 9.38 | -0.75 (-8.66- 7.16) | 21.62± 8.55 | -2.16 (-7.90- 3.58) |
| 30–39 | 15.44± 9.95 | -5.09 (-12.18- 1.99) | 19.58± 9.72 | -4.13 (-9.85- 1.59) |
| 40–49 | 16.69± 8.50 | -4.42 (-11.56- 2.72) | 21.37± 9.01 | -2.30 (-8.06- 3.45) |
| 50–59 | 17.58± 9.95 | -3.51 (-10.49- 3.47) | 21.31± 10.30 | -2.46 (-9.39- 4.46) |
| ≥60 | 21.33± 9.17 | Reference | 23.27± 9.81 | Reference |
| p-value | 0.086 | | 0.486 | |
| | | Marital status | | |
| Single | 17.44± 9.43 | 5.52 (-2.61- 13.67) | 21.37± 8.79 | -0.27 (-4.15- 3.59) |
| Married | 17.97± 9.49 | 6.81 (-1.08- 14.69) | 21.11± 9.13 | 0.23 (-3.41- 3.87) |
| Divorced/ widowed | 10.83± 9.51 | Reference | 20.89± 9.58 | Reference |
| p-value | 0.207 | | 0.922 | |
| | | Education | | |
| Basic education levels | 17.51± 9.89 | -1.24 (-5.15- 2.66) | 21.13± 9.02 | 1.14 (-1.90- 4.20) |
| Bachelor's degree /Higher diploma | 17.32± 9.15 | -1.48 (-5.17- 2.21) | 21.36± 8.94 | 1.47 (-1.20- 4.15) |
| Postgraduate | 19.06± 10.51 | Reference | 20.85± 9.00 | Reference |
| p-value | 0.729 | | 0.553 | |
| | | Occupation | | |
| Student | 17.55± 9.52 | -0.64 (-5.23- 3.94) | 21.50± 8.61 | 2.25 (-0.27- 4.78) |
| Government employed | 17.25± 9.82 | -1.39 (-6.55- 3.77) | 22.34± 9.15 | 4.62 (1.82- 7.42) |
| Private employed | 18.68± 9.71 | 0.06 (-5.31- 5.44) | 21.72± 8.69 | 2.06 (-1.95- 6.08) |
| Free work | 12.09± 5.44 | -6.30 (-13.76- 1.16) | 26.00± 6.50 | 6.16 (0.01- 12.32) |
| Retired | 17.93± 8.98 | -0.33 (-7.18- 6.52) | 24.30± 9.77 | 6.47 (-0.02- 12.97) |
| Unemployed | 18.15± 8.97 | Reference | 18.86± 9.24 | Reference |
| p-value | 0.495 | | **0.016** | |
| | | Monthly income | | |

| | | | | |
|---|---|---|---|---|
| <3,000 | 16.01± 8.71 | -1.72 (-5.23- 1.78) | 20.47± 9.34 | 0.13 (-3.37- 3.64) |
| 3,000–7,000 | 19.39± 9.32 | 1.60 (-1.89- 5.09) | 21.33± 8.23 | 0.76 (-2.47- 4.00) |
| 7,000–12,000 | 16.51± 9.76 | -1.16 (-4.28- 1.96) | 21.38± 9.16 | 0.71 (-2.57- 3.98) |
| 12,000–20,000 | 17.86± 9.87 | 0.07 (-2.78- 2.94) | 21.76± 9.21 | 1.09 (-2.04- 4.23) |
| >20,000 | 17.84± 8.90 | Reference | 20.42± 9.12 | Reference |
| p-value | 0.316 | | 0.944 | |

The p-values were analysed using univariate regression adjusted for age group, marital status, residence, education, occupation, and monthly income depending on the test fixed factor. CI: confidence interval

Table S7. Association between food sustainability attitude scores (continuous) and Saudi participants' socio-demographic characteristics, stratified by sex.

| | Male | | Female | |
|---|---|---|---|---|
| **Socio-demographic** | **Mean ± SD** | **Beta coefficients (95% CI)** | **Mean ± SD** | **Beta coefficients (95% CI)** |
| | | Age (years) | | |
| 18–29 | 3.60±1.93 | -1.00 (-2.58- 0.57) | 3.82±1.81 | --0.35 (-1.54- 0.83) |
| 30–39 | 4.54±1.79 | --0.22 (-1.64- 1.18) | 4.04±1.87 | -0.20 (-1.38- 0.98) |
| 40–49 | 4.33±1.98 | -0.41 (-1.83- 1.01) | 4.14±1.79 | -0.16 (-1.36- 1.02) |
| 50–59 | 4.71±1.84 | -0.16 (-1.55- 1.22) | 3.81±2.53 | -0.43 (-1.87- 1.00) |
| ≥60 | 4.78±1.98 | Reference | 4.18±1.60 | Reference |
| p-value | 0.286 | | 0.934 | |
| | | Marital status | | |
| Single | 3.72±1.94 | 0.09 (-1.53- 1.71) | 3.85±1.83 | -0.09 (-0.89- 0.71) |
| Married | 4.47±1.88 | 0.08 (-1.48- 1.65) | 4.06±1.82 | -0.05 (-0.80- -0.70) |
| Divorced/ widowed | 4.33±2.25 | Reference | 3.96±2.04 | Reference |
| p-value | 0.994 | | 0.972 | |
| | | Education | | |
| Basic education levels | 3.60±2.05 | -0.43 (-1.21- 0.33) | 3.75±1.82 | -0.36 (-0.99- 0.26) |
| Bachelor's degree /Higher diploma | 4.08±1.90 | 0.01 (-0.72- 0.74) | 3.95±1.87 | -0.21 (-0.76- 0.34) |
| Postgraduate | 4.38±1.82 | Reference | 4.11±1.73 | Reference |
| p-value | 0.108 | | 0.521 | |
| | | Occupation | | |
| Student | 3.54±1.91 | -0.59 (-1.50- 0.31) | 3.79±1.87 | 0.02 (-0.50- 0.55) |
| Government employed | 4.25±1.91 | -0.32 (-1.35- 0.69) | 4.21±1.82 | 0.22 (-0.35- 0.81) |
| Private employed | 4.82±1.78 | 0.33 (-0.73- 1.40) | 4.12±1.50 | 0.36 (-0.46- 1.21) |
| Free work | 4.36±2.29 | -0.21 (-1.69- 1.26) | 4.67±1.80 | 0.94 (-0.34- 2.21) |
| Retired | 4.59±1.95 | -0.24 (-1.60- 1.11) | 4.40±2.06 | 0.51 (-0.83- 1.86) |
| Unemployed | 4.16±1.74 | Reference | 3.77±1.83 | Reference |
| p-value | 0.184 | | 0.640 | |
| | | Monthly income | | |

| | | | | |
|---|---|---|---|---|
| <3,000 | 3.13±1.90 | -0.61 (-1.30- 0.07) | 3.77±1.93 | -0.61 (-1.34- 0.11) |
| 3,000–7,000 | 3.85±1.72 | 0.13 (-0.550 0.81) | 3.83±1.79 | -0.54 (-1.21- 0.11) |
| 7,000–12,000 | 3.68±1.95 | -0.18 (-0.79- 0.43) | 3.71±1.83 | -0.72 (-1.40- -0.04) |
| 12,000–20,000 | 4.61±1.67 | 0.65 (0.09- 1.21) | 4.09±1.84 | -0.37 (-1.02- 0.27) |
| >20,000 | 3.97±2.35 | Reference | 4.48±1.77 | Reference |
| p-value | **0.000** | | 0.269 | |

The p-values were analysed using univariate regression adjusted for age group, marital status, residence, education, occupation, and monthly income depending on the test fixed factor. CI: confidence interval

Table S8. Association between food waste behaviour scores (continuous) and Saudi participants' socio-demographic characteristics, stratified by sex.

| | **Male** | | **Female** | |
|---|---|---|---|---|
| **Socio-demographic** | **Mean ± SD** | **Beta coefficients (95% CI)** | **Mean ± SD** | **Beta coefficients (95% CI)** |
| | | Age (years) | | |
| 18–29 | 16.51±8.79 | 2.24 (-5.00- 9.48) | 15.11±8.60 | -4.65 (-9.95- 0.63) |
| 30–39 | 15.66±8.08 | 2.83 (-3.65- 9.32) | 15.78±7.96 | -2.56 (-7.84- 2.71) |
| 40–49 | 15.76±8.83 | 3.06 (-3.46- 9.60) | 14.34±7.65 | -3.65 (-8.96- 1.64) |
| 50–59 | 12.63±8.74 | 0.40 (-5.98- 6.79) | 14.19±7.07 | -4.09 (-10.48- 2.29) |
| ≥60 | 12.11±9.04 | Reference | 18.55±7.09 | Reference |
| p-value | 0.593 | | 0.323 | |
| | | Marital status | | |
| Single | 16.43±8.59 | 1.14 (-6.27- 8.56) | 15.34±8.44 | 2.71 (-0.85- 6.28) |
| Married | 14.64±9.00 | 2.01 (-5.18- 9.19) | 15.14±7.78 | 2.49 (-0.85- 5.85) |
| Divorced/ widowed | 12.83±7.62 | Reference | 13.14±8.78 | Reference |
| p-value | 0.764 | | 0.293 | |
| | | Education | | |
| Basic education levels | 17.37±8.52 | 1.15 (-2.39- 4.69) | 15.48±8.46 | 2.15 (-0.65- 4.97) |
| Bachelor's degree /Higher diploma | 15.17±8.71 | -0.95 (-4.30- 2.38) | 15.22±8.36 | 2.08 (-0.37- 4.55) |
| Postgraduate | 15.34±9.30 | Reference | 14.04±7.06 | Reference |
| p-value | 0.099 | | 0.233 | |
| | | Occupation | | |
| Student | 16.62±8.71 | 1.57 (-2.59- 5.74) | 15.62±8.49 | 1.46 (-0.87- 3.81) |
| Government employed | 16.02±9.03 | 3.61 (-1.08- 8.30) | 13.86±7.36 | -0.11 (-2.70- 2.47) |
| Private employed | 14.91±9.15 | 1.95 (-2.92- 6.84) | 15.96±7.20 | 1.29 (-2.41- 5.01) |
| Free work | 11.45±8.85 | -1.13 (-7.91- 5.64) | 19.33±8.17 | 4.67 (-1.01- 10.36) |
| Retired | 13.14±7.01 | 1.54 (-4.68- 7.77) | 18.80±7.17 | 2.97 (-3.03- 8.99) |
| Unemployed | 14.53±8.01 | Reference | 14.44±8.66 | Reference |
| p-value | 0.394 | | 0.422 | |
| | | Monthly income | | |

| | | | | |
|---|---|---|---|---|
| <3,000 | 17.42±9.17 | 1.60 (-1.60- 4.81) | 15.72±8.64 | 2.05 (-1.15- 5.26) |
| 3,000–7,000 | 17.21±9.01 | 1.31 (-1.87- 4.49) | 14.65±8.19 | 0.63 (-2.32- 3.60) |
| 7,000–12,000 | 15.59±8.51 | 0.01 (-2.83- 2.86) | 16.78±7.64 | 2.84 (-0.16- 5.85) |
| 12,000–20,000 | 14.88±8.27 | -0.36 (-2.98- 2.25) | 14.49±8.22 | 0.74 (-2.12- 3.62) |
| >20,000 | 15.16±9.16 | Reference | 13.57±8.74 | Reference |
| p-value | 0.614 | | 0.176 | |

The p-values were analysed using univariate regression adjusted for age group, marital status, residence, education, occupation, and monthly income depending on the test fixed factor. CI: confidence interval

Table S9. Socio-demographic factors associated with tertiles of food sustainability attitude scores among Saudi participants.

| Socio-demographic Characteristics | Attitudes | | | | |
|---|---|---|---|---|---|
| | First tertile (Lowest) (n = 202) n (%) | Second tertile (Middle) (n = 262) n (%) | Third tertile (Highest) (n = 391) n (%) | $X^2$ | p-value |
| | | Sex | | | |
| Male | 100 (50) | 111 (42) | 194 (50) | 3.792 | 0.150 |
| Female | 102 (50) | 151 (58) | 197 (50) | | |
| | | Age (years) | | | |
| 18–29 | 128 (63) | 178 (68) | 201 (51) | 26.011 | **0.001** |
| 30–39 | 31 (15) | 37 (14) | 76 (19) | | |
| 40–49 | 28 (14) | 33 (12) | 66 (17) | | |
| 50–59 | 13 (7) | 7 (3) | 37 (10) | | |
| ≥ 60 | 2 (1) | 7 (3) | 11 (3) | | |
| | | Marital status | | | |
| Single | 130 (64) | 181 (69) | 221 (56) | 11.100 | **0.025** |
| Married | 64 (32) | 73 (28) | 152 (39) | | |
| Divorced/widowed | 8 (4) | 8 (3) | 18 (5) | | |
| | | Education | | | |
| Basic education levels | 61 (30) | 66 (25) | 85 (22) | 6.419 | 0.170 |
| Bachelor's degree /Higher diploma | 126 (63) | 167 (64) | 263 (67) | | |
| Postgraduate | 15 (7) | 29 (11) | 43 (11) | | |
| | | Occupation | | | |
| Student | 111 (55) | 145 (55) | 159 (41) | 34.139 | **0.000** |
| Government employ | 41 (20) | 47 (18) | 103 (26) | | |
| Private employ | 10 (5) | 15 (6) | 44 (12) | | |
| Free work | 4 (2) | 3 (1) | 13 (3) | | |
| Retired | 8 (4) | 7 (3) | 24 (6) | | |
| Unemployed | 28 (14) | 45 (17) | 48 (12) | | |

| | | | | | |
|---|---|---|---|---|---|
| | | Monthly income | | | |
| < 3,000 | 40 (20) | 46 (18) | 43 (11) | | |
| 3,000–7,000 | 41 (20) | 64 (24) | 71 (18) | | |
| 7,000–12,000 | 53 (26) | 66 (25) | 69 (18) | 38.946 | **0.000** |
| 12,000–20,000 | 44 (22) | 63 (24) | 149 (38) | | |
| > 20,000 | 24 (12) | 23 (9) | 59 (15) | | |

Differences between groups were assessed using the chi-square test; $p < 0.05$ was considered significant.

Table S10. Intake of selected food items according to tertiles of food sustainability attitude scores

| **Food Item** | **Low Score (n = 202)** | **Medium Score (n = 262)** | **High Score (n = 391)** |
|---|---|---|---|
| | | Legumes | |
| Mean ± SD | 1.33 ± 1.05 | 1.47 ± 1.05 | 1.63 ± 1.01 |
| Beta coefficients (95% CI) | −0.27− (−0.45−to −0.09−) | −0.13− (−0.30−to 0.02) | Reference |
| Adjusted p-value | | **0.009** | |
| | | Fish | |
| Mean ± SD | 1.04 ± 0.86 | 1.03 ± 0.86 | 1.14 ± 0.87 |
| Beta coefficients (95% CI) | −0.05− (−0.20− 0.09) | −0.06− (−0.20− 0.07) | Reference |
| Adjusted p-value | | 0.588 | |
| | | Pasta | |
| Mean ± SD | 1.74 ± 1.03 | 1.82 ±0.98 | 1.88 ± 0.93 |
| Beta coefficients (95% CI) | −0.14− (−0.30− 0.02) | −0.06− (−0.22− 0.08) | Reference |
| Adjusted p-value | | 0.251 | |
| | | Red meat | |
| Mean ± SD | 1.51 ± 1.04 | 1.89 ± 1.17 | 2.14 ± 1.11 |
| Beta coefficients (95% CI) | −0.57− (−0.76− to −0.38−) | −0.17− (−0.35− to 0.001) | Reference |
| Adjusted p-value | | **0.000** | |
| | | White meat | |
| Mean ± SD | 2.00 ± 1.17 | 2.37 ± 1.25 | 2.77 ± 1.07 |
| Beta coefficients (95% CI) | −0.70− (−0.90− to −0.51−) | −0.33− (−0.52− to −0.15−) | Reference |
| Adjusted p-value | | **0.000** | |
| | | Processed meat | |
| | 0.94 ± 1.09 | 0.79 ± 1.06 | 0.84 ± 1.16 |
| Beta coefficients (95% CI) | 0.08 (−0.10− 0.28) | −0.05− (−0.23− 0.12) | Reference |
| Adjusted p-value | | 0.398 | |
| | | Eggs | |
| Mean ± SD | 2.08 ± 1.18 | 2.38 ± 1.24 | 2.73 ± 1.20 |
| Beta coefficients (95% CI) | −0.58− (−0.79− to −0.38−) | −0.29− (−0.48− to −0.10−) | Reference |
| Adjusted p-value | | **0.000** | |
| | | Milk | |
| Mean ± SD | 2.11 ± 1.26 | 2.43 ± 1.24 | 2.60 ± 1.25 |
| Beta coefficients (95% CI) | −0.44− (−0.65− to −0.22−) | −0.12− (−0.32− to 0.08) | Reference |

| | | | |
|---|---|---|---|
| Adjusted p-value | | **0.000** | |
| | | Yogurt | |
| Mean ± SD | 2.07 ± 1.18 | 2.31 ± 1.22 | 2.57 ± 1.15 |
| Beta coefficients (95% CI) | −0.44− (−0.64− to −0.23−) | −0.21− (−0.40− to −0.03−) | Reference |
| Adjusted p-value | | **0.000** | |
| | | Cheese | |
| Mean ± SD | 2.19 ± 1.21 | 2.51 ± 1.22 | 2.69 ± 1.24 |
| Beta coefficients (95% CI) | −0.45− (−0.66− to −0.24−) | −0.14− (−0.34− to 0.05) | Reference |
| Adjusted p-value | | **0.000** | |
| | | Vegetables | |
| Mean ± SD | 2.14 ± 1.24 | 2.55 ± 1.25 | 2.72 ± 1.24 |
| Beta coefficients (95% CI) | −0.54− (−0.75− to −0.33−) | −0.14− (−0.34− to 0.04) | Reference |
| Adjusted p-value | | **0.000** | |
| | | Fruits | |
| Mean ± SD | 2.01 ± 1.16 | 2.31 ± 1.20 | 2.45 ± 1.24 |
| Beta coefficients (95% CI) | −0.39− (−0.59− to −0.18−) | −0.12− (−0.31− to 0.07) | Reference |
| Adjusted p-value | | **0.001** | |
| | | Bread | |
| Mean ± SD | 2.50 ± 1.40 | 2.78 ± 1.26 | 3.12 ± 1.31 |
| Beta coefficients (95% CI) | −0.55− (−0.78− to −0.32−) | −0.27− (−0.48− to −0.06−) | Reference |
| Adjusted p-value | | **0.000** | |
| | | Sweet or savoury snacks | |
| Mean ± SD | 2.16 ± 1.25 | 2.20 ± 1.21 | 2.49 ± 1.33 |
| Beta coefficients (95% CI) | −0.30− (−0.52− to −0.08−) | −0.30− (−0.50− to −0.10−) | Reference |
| Adjusted p-value | | **0.003** | |
| | | Sweetened soft drinks | |
| Mean ± SD | 1.81 ± 1.32 | 1.75 ± 1.29 | 1.96 ± 1.48 |
| Beta coefficients (95% CI) | −0.15− (−0.39− 0.08) | −0.25− (−0.47− −0.03−) | Reference |
| Adjusted p-value | | 0.064 | |
| | | Low-calorie soft drinks | |
| Mean ± SD | 1.51 ± 1.25 | 1.50 ± 1.30 | 1.34 ± 1.34 |
| Beta coefficients (95% CI) | 0.10 (−0.11− 0.33) | 0.08 (−0.12− 0.29) | Reference |
| Adjusted p-value | | 0.570 | |

The p-value was analysed using univariate regression adjusted for sex, age group, marital status, education, occupation, and monthly income; $p < 0.05$ was considered significant. CI: confidence interval
